# Structured engineering of self-emulsifying drug delivery systems (SEDDS) *via* 3D printing: Comprehensive review

**DOI:** 10.1016/j.ijpx.2025.100416

**Published:** 2025-10-15

**Authors:** Induja Govindan, Anjana A. Kailas, K.A. Abutwaibe, Thamizharasan Annadurai, Ujwala Achar, Anup Naha, Srinivas Hebbar

**Affiliations:** Department of Pharmaceutics, Manipal College of Pharmaceutical Sciences, Manipal Academy of Higher Education, Manipal 576104, India

**Keywords:** 3D printing, Self-emulsifying drug delivery system, Personalised medicines, Polypharmacy, Combination medicines

## Abstract

The combination of self-emulsifying drug delivery systems (SEDDS) with three-dimensional printing (3DP) technologies represents an innovative and promising strategy for developing personalised dosage forms. Through precise control over dosage form architecture, controlled drug release kinetics, and individualised therapeutic customisation, 3DP offers distinct and transformative advantages over conventional pharmaceutical formulation approaches. This review focuses on the application of modern 3DP techniques, specifically fused deposition modelling (FDM), semi-solid extrusion (SSE), and drop-on-demand (DoD), in the formulation and production of SEDDS. Each technique is critically evaluated in terms of formulation compatibility, operational mechanisms, and its potential to address the current manufacturing challenges associated with SEDDS. 3DP technologies offer several benefits, including enhanced flexibility in production, the ability to fabricate on demand, and the potential to accommodate complex and personalised therapeutic regimens. However, these methods also face notable limitations, such as material constraints, variability in print quality, mechanical and safety issues, and a lack of clear regulatory guidance. Despite their potential, the use of 3DP in SEDDS development remains relatively unexplored. This review aims to provide a comprehensive overview of the current research landscape, identify existing technological and regulatory barriers, and discuss future prospects for integrating 3DP into next-generation SEDDS formulations.

## Highlights

• Provides a pioneering review on combining 3D printing of SEDDS.
• Critically analyses 3DP techniques (FDM, PAM/SSE, DoD) for formulation compatibility and potential.
• Highlights benefit for personalised dosing, combination therapy, and controlled release.
• Identifies the limitations: material constraints, print variability, and regulatory gaps.
• Outlines the future research directions to advance 3D-printed SEDDS.

## Graphical abstract

**Figure:**
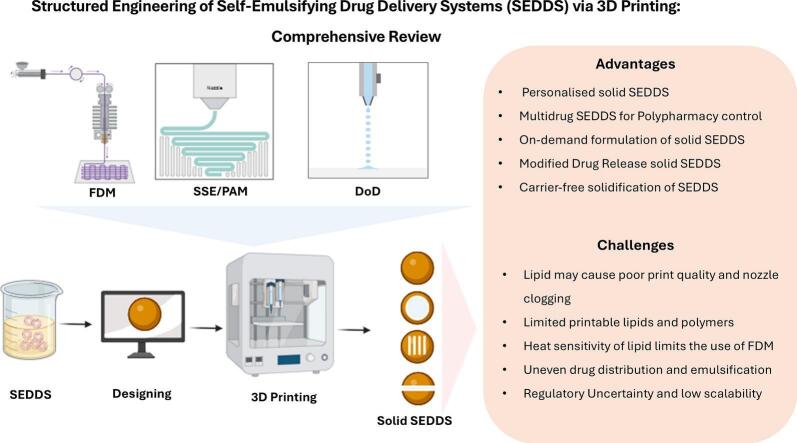

## 1 Introduction

Lipid-based formulations have the potential to significantly improve the oral bioavailability of poorly water-soluble drugs *via* different processes, including preserving the drug in a solubilised state, modifying gastric emptying, and altering post-absorption drug distribution (Čerpnjak et al., 2013) (Porter et al., 2007). Enzymatic digestion of lipids leads to the formation of digestion products such as monoglycerides and free fatty acids. These digestion products interact with endogenous components (*i.e.*, bile salts, phospholipids, and cholesterol) and form various colloidal structures that can act as transport media for the absorption of lipophilic drugs (Phan et al., 2014). Self-emulsifying drug delivery systems (SEDDS) have garnered considerable attention recently owing to their ability to solubilise poorly water-soluble drugs into finely dispersed oil droplets. SEDDS are isotropic combinations of oils, surfactants, and co-solvents/surfactants that generate fine, kinetically stable oil-in-water (o/w) emulsions with small particle sizes in the gastrointestinal tract (GIT) upon aqueous dilution (Abdalla et al., 2008; Agrawal et al., 2015; Abou Assi et al., 2020). Currently marketed SEDDS are primarily liquid-based and encapsulated in hard or soft gelatin capsules (Tran and Park, 2021). The commercialisation of SEDDS remains limited due to inherent challenges, including chemical stability and portability concerns, dose variation, softening of capsule shell and leakage, precipitation during dilution, high surfactant levels causing gastrointestinal irritation, and lack of *in vitro* evaluation models (Krstić et al., 2018; Buya et al., 2020; Khan et al., 2012). Consequently, there has been growing interest in transforming these liquid SEDDS into solid SEDDS (S-SEDDS) as a strategy to address some of these challenges.

Solidification of SEDDS enhances their physicochemical stability (Ghosh et al., 2020). Additionally, this approach offers an extended gastric residence time (Hussain et al., 2019), accurate dosing, reduced precipitation, ease of handling and good patient compliance (Chavan et al., 2015). The transformation of liquid SEDDS into a solid form can reduce surfactant-induced toxicity by minimising the surfactant content (Morozowich and Gao, 2009) and enhancing the oxidative stability of lipids by protecting them from degradation (Joyce et al., 2019). S-SEDDSs are conventionally prepared using various solidification strategies, including Physical Adsorption (Gumaste et al., 2013), spray drying (Kumar et al., 2023), spray congealing (Albertini et al., 2015), melt granulation (Passerini et al., 2006), supercritical fluid-based techniques (Reverchon et al., 2015), and melt extrusion/extrusion spheronization (Kulkarni et al., 2024). These methods generally require the incorporation of a substantial quantity of solid-phase carriers such as silica (Aerosil, Syloid, Sylysia, Neusilin), carbohydrates (dextran, lactose, Mannitol, Lactose), and crosslinked polymers (microcrystalline cellulose, Avicel) to effectively encapsulate the liquid SEDDS and develop an S-SEDDS (Govindan et al., 2024). The use of solid carriers can also present challenges, such as dose dilution, tolerability concerns, and potential toxicity. Additionally, the reliance on large amounts of solid carriers may affect the overall bioavailability and stability of SEDDS, limiting its effectiveness in clinical applications (Katteboina and P, 2009). Therefore, it would be ideal to find an alternative strategy for developing S-SEDDSs that does not require a high concentration of solid carriers.

3D printing (3DP) has shown great promise and has attracted considerable interest across various disciplines, such as material science and drug development (Holländer et al., 2016) (Boetker et al., 2016) (Melocchi et al., 2015). Charles Hull was the driving force behind the invention of 3D printing, also known as additive manufacturing (AM), rapid prototyping (RP), or solid-freeform technology (SFF) (Chapman, 2022). 3D printing is commonly used for rapid prototyping of 3D models designed using computer-aided design (CAD) software (AutoDesk or AutoCAD). Once the model is created in the CAD program, it is converted into a Standard Tessellation Language (STL) or Stereolithography file. The STL format is widely recognized as the standard for transferring design data from computer-aided design (CAD) software to 3D printers. This file format represents the surfaces of a 3D model as a series of triangular facets with vertex coordinates recorded in a text file. The text file contains extra data points to spatially define the part surface when the number of triangles defining the surface is increased. The printed material has a higher resolution because of the increased vertices (Gross et al., 2014).

The pharmaceutical industry is witnessing a worldwide surge in research on 3D printing technology. The FDA (Food and Drug Administration, United States) approved the first 3D printed orodispersible tablet, Spritam® (levetiracetam), in 2015, confirming the potential of 3DP in the development and manufacturing of pharmaceutical solid dosage forms (Fitzgerald, 2015). Compared to conventional preparation methods, 3D printing offers exceptional versatility for developing intricate 3D drug structures, customised dosage forms with exceptional creativity and flexibility, as well as rapid prototyping and production. 3DP offers precise control over drug release and meets a wide range of clinical requirements. Furthermore, it drastically reduces the development period by transforming drug manufacturing methods and redefining the design, manufacture, and consumption (Jacob et al., 2020).

However, research on 3DP has predominantly focused on polymer-based drug delivery systems with considerably less information available on lipid-drug delivery systems. The application of 3DP technologies to adjust and refine the specific properties of lipid drug delivery systems is promising because lipids are soft materials that melt at low temperatures. Lipids are beneficial thermolabile compounds that can be processed at low temperatures. The crystalline or amorphous structure of the drug also remains intact at low processing temperatures, which may help maintain the stability of the drug. Furthermore, lipids can improve the solubility of lipophilic drugs, which may increase the possibility of producing formulations with high drug loadings (Vithani et al., 2019b). To the best of our knowledge, only a few studies using 3DP technology have been published for SEDDS. The objective of this review is to present a multifaceted analysis of different 3D printing technologies, outline the current state of their use and manufacturing principles in the field of SEDDS solidification, and elucidate the benefits and drawbacks of each technology, as well as the appropriate pharmaceutical dosage forms that should be produced.

## 2 Self-emulsifying drug delivery system

Self-emulsifying drug delivery systems (SEDDS) are characterised as isotropic blends comprising natural or synthetic oils, solid or liquid surfactants, and one or more hydrophilic solvents or co-solvents/co-surfactants (Gursoy and Benita, 2004). Upon gentle mixing followed by dilution in aqueous media, these systems spontaneously generate fine oil-in-water emulsions. The term SEDDS generally describes formulations that create emulsions with droplet sizes ranging from a few nanometers to several micrometres. Self-microemulsifying drug delivery systems (SMEDDS) are systems that form clear microemulsions with droplet sizes ranging between 100 and 250 nm. A newer term, the self-nanoemulsifying drug delivery system (SNEDDS), defines formulations that produce nanoemulsions with droplet sizes below 100 nm (Kohli et al., 2010).

The interaction of the components, oil, and surfactants results in self-emulsification. The type of oil and surfactant, as well as their ratios, must be carefully selected to create an effective self-emulsifying system. Adequate solubility in lipids and good self-emulsification efficiency of surfactants and cosurfactants are desirable for active medicinal agents.

The key component that significantly contributes to the development of SEDDS is oil. Long- and medium-chain triglyceride (TG) oils with different saturation levels are commonly used. Natural oils (castor, lemon, olive, and clove oils) containing medium-chain triglycerides are usually not recommended because of their limited capacity to dissolve significant quantities of lipophilic drugs. Modified long- and medium-chain triglyceride oils (Campul MCM, Labrafil M 1944 CS, Imwitor, *etc.*) with different levels of saturation have been widely employed for the development of SEDDS. When combined with numerous solubility-enhancing surfactants approved for oral administration, these semisynthetic compounds create effective emulsification systems (Dokania and Joshi, 2015) (Govindan et al., 2024). It has been discovered that, in contrast to medium-chain lipids, long-chain lipids can sustain drug levels without precipitation (Prasad et al., 2013). Oils improve the solubility of lipophilic drugs and their lymphatic system delivery.

The amphiphilic nature of surfactants enables them to solubilise significant amounts of lipophilic drugs in the gastrointestinal lumen without precipitating. The suitability of the surfactant for the formulation of SEDDS is determined by its Hydrophilic-Lipophilic Balance (HLB) value. Surfactants with high HLB values can achieve a high emulsifying performance, facilitating the rapid creation of oil-in-water droplets (Kohli et al., 2010). Natural surfactants (Eg, Lecithin) are generally preferred over synthetic materials because of their favourable safety profile and excellent biocompatibility. However, their self-emulsifying performance is inherently limited (Nardin and Köllner, 2019). Gelucire® 50/13, Gelucire® 44/14, Tween 20, Tween 80, Cremophor® EL, Cremophor® RH 40, and Labrasol® are among the most suitable non-ionic surfactants for SEDDS formulations, primarily because of their lower hazard potential compared to ionic surfactants. Moreover, nonionic surfactants stabilised the emulsion formed. The cytotoxicity and permeability of surfactants must be considered when formulating SEDDS formulations because high concentrations of these surfactants can irritate the gastrointestinal tract (Maji et al., 2021).

A relatively high concentration of surfactants was used to solubilise significant quantities of drugs. Co-surfactants play a critical role in enhancing the solubility of hydrophilic surfactants and drugs within the lipid matrix. Moreover, they improved the interfacial fluidity between the two phases of the emulsion and optimised the emulsification process to achieve superior system stability and performance (Govindan et al., 2024). Transcutol HP, ethanol, isopropanol, Polyethylene glycol (PEG) 200, PEG 400, and Span 20 are commonly used as cosurfactants (Gao et al., 2021).

Currently marketed SEDDS (Neoral, Sandimmune, Targretin, Accutane) are primarily liquid-based and encapsulated in hard or soft gelatin capsules (Tran and Park, 2021). The commercialization of SEDDS formulations remains limited due to inherent challenges, including chemical stability and portability concerns, dose variation, softening of capsule shell and leakage, precipitation risks during dilution, high surfactant levels causing gastrointestinal irritation, and lack of *in vitro* evaluation models (Krstić et al., 2018) (Buya et al., 2020). Consequently, there has been growing interest in transforming these liquid SEDDS into solid SEDDS (S-SEDDS) as a strategy to address some of these challenges. Conventional solidification methods (spray drying, physical adsorption, lyophilization, spray congestion, *etc.*) depend on large amounts of solid carriers to develop solid SEDDS, which may affect the overall bioavailability and stability of SEDDS, limiting its effectiveness in clinical applications (Katteboina and P, 2009). Therefore, it would be ideal to employ 3D printing technology to develop S-SEDDSs because it does not require a high concentration of solid carriers.

## 3 Insights into 3D printing

Over the past ten years, economic growth, globalisation, and industrialisation have greatly increased the importance of the manufacturing sector. Several innovative approaches have been introduced, highlighting the fact that innovation is an ongoing process. The recent explosion in 3D printing has destroyed traditional norms.

Digitally sophisticated software such as Onshape, Solid Works, Creo Parametric, Autocad, Autodesk Tinker cad, BRL-CAD, Free CAD, Open SCAD, Wings3D, 3D Slash, Sketch UP, and Fusion 360 are used to create a virtual 3D design of an object. This sophisticated model was then converted into a digital file format (.STL), which is a standard tesselation language or stereolithography, using MeshLab, Google SketchUp, a plugin, STL-viewer, and Netfabb Studio software. The CAD file specifies the dimensions and geometry of the components that must be constructed. Triangular coordinates that collectively comprise the surface of the intended 3D design are provided in an STL format file (Goole and Amighi, 2016). The process of converting a 3D model into a series of flat layers is known as slicing. Software such as MatterControl, Ultimaker Cura, Slic3r, OctoPrint, and Idea Maker is commonly used for slicing. Slicing software converts a 3D design into multiple 2D layers, guiding the printer's extruder, laser, or similar fixation methods. This process transforms a standard tesselation language (.STL) file into a G-code file using a specialised slicer software. After slicing, an appropriate printing material was selected. 3D printers use filaments as their “ink,” which can be made from a variety of materials, including plastics, ceramics, resins, metals, textiles, biomaterials, glass, food, and even lunar dust. The computer instructs the 3D printer to deposit material layer-by-layer after the model has been loaded. A 3D printer operates by extruding molten plastic through a tiny nozzle. The printer waits for the first layer to dry before printing the subsequent layer on top. This cycle was repeated until the desired product was obtained (Mohapatra et al., 2022).

In biomanufacturing, 3D printing uses scaffold-based engineering to promote bone and tissue regeneration, while in the pharmaceutical sector, it is used for both drug discovery and the development of drug delivery systems (Do et al., 2015). The advent of 3D printing technology has provided an important tool for developing nanoformulations into solid dosage forms, particularly in the form of oral 3D printed tablets (Sen et al., 2020), as well as suppositories (Wei et al., 2023) for the local and systemic distribution of poorly soluble medications. The nanoformulations were successfully built into a strong solid dosage form using 3D printing technology without compromising the nanonization properties of the loaded pharmaceuticals or therapies. The application of 3D printing in formulation optimisation enables the production of patient-centred drug products, such as chewable and flavored medications, to improve adherence among pediatric and elderly patients (Krause et al., 2021) (Hanning et al., 2016).

## 4 3D printing technology to design SEDDS: recent advances

Integrating 3D printing technologies with SEDDS provides a novel avenue for developing personalised solid SEDDS. Vithani et al. showed a proof of concept that a liquid self-emulsifying formulation system can be transformed into patient-specific oral solid dosage forms *via* 3D printing without solid carriers by developing S-SMEDDS of fenofibrate or cinnarizine, composed of Gelucire® 44/14, Gelucire® 48/16 and Kolliphor® P 188 (Vithani et al., 2019a).

Various 3D printing methods can be tailored to handle the unique characteristics of SEDDS, such as their liquid or semi-solid nature, the requirement for solidification, and the need to maintain emulsification properties. The key difference between 3DP technologies is the method of forming and assembling distinct layers of materials to generate the final object (Madla et al., 2018).

The 3DP technology chosen determines the choice of feed materials. Furthermore, the characteristics of the drug, such as the melting point and degradation temperature, affect the selection of the 3DP technique, which further affects the suitability of the materials and printing procedures. The broadly recognized classification of 3D printing technologies encompasses: (i) inkjet printing-based methods, including continuous inkjet printing (CIJ) and drop-on-demand (DOD) printing; (ii) laser-based techniques, such as stereolithography (SLA), selective laser sintering (SLS), and Digital Micromirror Device (DMD) technology; and (iii) nozzle-based deposition methods, including fused deposition modelling (FDM) and pressure-assisted microsyringe (PAM) techniques (Pérez Gutiérrez et al., 2023).

Here, we compiled 3DP technologies compatible with SEDDS solidification, including fused deposition modelling (FDM), Pressure-Assisted Microextrusion (PAM), and drop-on-demand (DOD).

### 4.1 Material extrusion methods

#### 4.1.1 Fused deposition modelling (FDM)

Fused deposition modelling (FDM) is the most popular and economical 3D printing method (Patel et al., 2022). In this procedure, filament-shaped thermoplastic polymers were extruded through a heated printer nozzle to create the desired structure (Okafor-Muo et al., 2020). The material is dispensed along a predetermined path at controlled temperatures, depositing semi-molten layers onto the construction platform (Kristiawan et al., 2021). The core principle of FDM manufacturing involves heating the raw material until it softens, reshaping it, and feeding it into a temperature-regulated nozzle that maintains a semisolid state. The filament, supplied by a spool, is drawn by a drive wheel and extruded precisely to form ultrathin layers. These layers are deposited sequentially, guided by the contours defined in a program, typically a CAD model integrated into the FDM system, enabling the construction of structural elements one layer at a time (Dizon et al., 2018) Fig. 1. The use of elevated temperatures during extrusion and printing, difficulty in producing filaments with suitable mechanical and viscoelastic properties, and slow printing speed are some limitations of this technique. Nevertheless, it remains widely favoured owing to its cost-effectiveness, reliance on non-volatile materials, simplicity, and versatility (Norman et al., 2017).

**Fig. 1:**
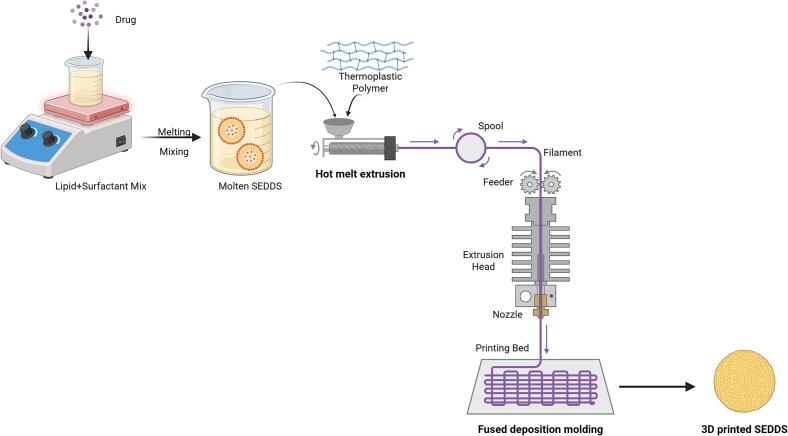
Schematic representation of the fabrication of 3D-printed self-emulsifying drug delivery systems (SEDDS) using fused deposition modelling (FDM).

Polylactic acid (PLA), polylactide-coglycoside (PLGA), polyvinyl alcohol (PVA), polycaprolactone (PCL), and other cellulose derivatives are frequently used filament materials. The most popular way to create drug-loaded filaments is the passive soaking method, in which filament materials are immersed in a drug-containing saturated methanol or ethanol solvent, followed by a thorough drying process (Cui et al., 2021). A simple and reliable hot melt extrusion (HME) process can also be used to produce drug-loaded polymeric filaments with specific excipients. A recently developed technology for the 3D printing of pharmaceuticals combines the HME and FDM. HME and FDM can be used to create customised medication products with predetermined dosages. Furthermore, downstream processing involving traditional dosage forms is decreased by this combination technique (Dumpa et al., 2021) (Bandari et al., 2021). Furthermore, if a 3D printer is designed to use the polymer and drug directly in their raw form, the drug-loading filament preparation step can be eliminated (Shaqour et al., 2020).

##### 4.1.1.1 Case study 1

**Fig. 2:**
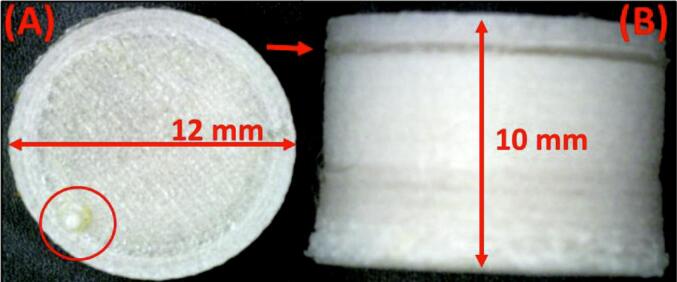
Microscopical image of container tablet: (A) top view and (B) side view. Figure reproduced with permission from (Kulkarni et al., 2022).

The integration of 3D printing and SNEDDS has recently demonstrated a promising approach to overcome the inherent challenges of multi-drug delivery and stabilisation of drugs susceptible to degradation under acidic gastric conditions. Kulkarni, V. R. demonstrated the application of FDM 3DP to develop a bioactive self-nano-emulsifying hollow container tablet for controlled delivery of multiple drugs (Curcumin and Lansoprazole). They explored FDM 3D-printing technology to design hollow tablets with a variety of forms that resemble containers. The composition of the printing filament is the key parameter for customising the controlled release of the drug. Super-disintegrants and hydrophilic polymers, such as hydroxypropyl cellulose, facilitate rapid drug release compared to pH-dependent polymers such as Hydroxypropyl Methylcellulose Acetyl Succinate grades, which swell to create a matrix-resembling hydrogel. The cylindrical model of the hollow tablet containers was designed using SolidWorks (Dassault Systèmes, Vélizy-Villacoublay, France), and an FDM 3D printer (Ultimaker S3, Utrecht, The Netherlands) was used to print the hollow tablet containers. Before testing, the ideal printing temperature was determined, starting at the maximum extrusion temperature, to prevent nozzle clogging. The release of SNEDDS from the hollow container tablet was prolonged by modulating the thickness of the walls, achieving lag phases of 30 and 60 min for wall thicknesses of 0.4 mm and 1 mm, respectively (Fig. 2). The study shows that the floating property and delayed release of 3D-printed hollow tablets enable sustained SNEDDS delivery, overcoming the limitation of uncontrolled release seen with conventional formulations (Kulkarni et al., 2022).

A clear innovation by using FDM-based hollow container tablets for multi-drug delivery and protection against gastric degradation, highlighting the versatility of design achievable through the 3DP technique. This study establishes a direct relationship between formulation parameters and performance outcomes, such as the influence of excipient selection on release kinetics and the dual functional advantage of floating ability combined with delayed drug release. The modulation of wall thickness provided measurable control over lag phases, demonstrating reproducibility and a strong structure-performance correlation. Process optimisation, including extrusion temperature adjustment, further supports the robustness of the approach. However, while these findings establish solid proof of concept, the evaluation is limited by the absence of *in-vivo* pharmacokinetic validation and exploration of broader design parameters beyond wall thickness, which are essential to confirm the translational impact of this promising platform.

##### 4.1.1.2 Case study 2

**Fig. 3:**
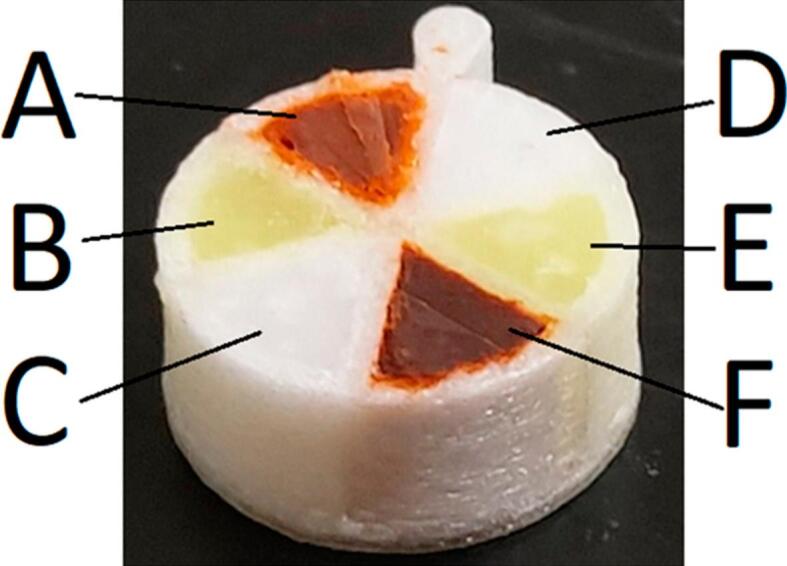
Representative 3D-printed multicompartment Polymer-Lipid Hybrid (PLH) Tablet prepared from poly-vinyl-alcohol, each loaded with six combinations of clofazimine, lumefantrine, and halofantrine in two different solid SMEDDS formulations. Figure reproduced with permission from: (Barber et al., 2021).

In a recent study, 3D-printed single- and multi-compartment polymer scaffolds filled with dispersible SMEDDS formulations were developed, and their controlled dispersion and drug release were evaluated. Single-compartment polymer scaffolds with varying surface areas were 3D-printed and filled with a solid SMEDDS formulation containing the poorly soluble fenofibrate. Scaffolds are made from polylactic acid, which is resistant to gut degradation, or polyvinyl alcohol, which dissolves over physiological timescales. They also designed and printed a multi-compartment scaffold with six segmented wedges, each loaded with unique combinations of clofazimine, lumefantrine, and halofantrine in two different solid SMEDDS formulations, demonstrating precise, multi-drug delivery capabilities (Fig. 3). Physical scaffolds were created using commercial polylactic acid and polyvinyl alcohol filaments in an FDM-based 3D printer. During the design process, the scaffolds were modelled and exported as stereolithography files using the CAD software. Slicing software with personalised profiles was used to process the files to prepare them for printing. The final instructions were manually chosen for printing after being stored as G-code files on secure digital cards. The build plate and nozzle were preheated to ensure a smooth filament extrusion.

Regardless of the drug used, the findings showed that the surface-area-to-volume ratio (SA: V) reliably controlled dispersion and drug release. While formulations with a zero SA: V ratio exhibited neither dispersion nor drug release, formulations with the highest SA: V ratio achieved 90 % to 110 % dispersion and complete drug release in 20 min. These findings demonstrate the possibility of developing novel dosage forms with substantial advantages, especially in the management of polypharmacy, by optimising SA: V to regulate drug release profiles and enhance therapeutic efficacy (Barber et al., 2021).

The study shows strong evaluation by linking scaffold design to performance, demonstrating that surface-area-to-volume ratio reliably controls dispersion and release, with up to 90–110 % dispersion and complete drug release in 20 min. The use of PLA and PVA scaffolds and multi-compartment loading highlights innovation for polypharmacy, while the clear structure–function correlation strengthens reproducibility, though *in vivo* validation is still required.

The practical translation of this concept depends on developing faster printing processes. While dual-nozzle 3D printers make combination dosage forms technically feasible, production speed is influenced by material and method rather than design complexity. Currently, such formulations are better suited for niche, personalised use in clinical settings rather than large-scale manufacturing, though future advances may enable broader production (Tan et al., 2018).

#### 4.1.2 Pressure-assisted microextrusion (PAM)

Pressure-assisted microextrusion is also known as semi-solid extrusion (SSE). The technology uses a syringe filled with a gel or paste, such as FDM, but without filaments or powder. PAM generates more complex structures than other 3DP methods (Mohammed et al., 2021). The initial ingredients, usually pastes or gels, are prepared by mixing the optimum amount of chemicals with solvents to produce a viscosity suitable for printing. Mechanical, chemical, and physical properties such as viscosity, rheological behaviour, and material miscibility can significantly impact the processing results (Vithani et al., 2019b). The ease of extrusion and the capacity to maintain structural integrity after extrusion are the two key factors that establish the eligibility of a material for extrusion-based 3D printing. The material satisfies the requirements to be optimised as a suitable material for applications because it offers printed 3D object stability and easy deposition. The material can be extruded through a printer nozzle by exerting pressure. The rheological behaviour of the material and the size and shape of the printer nozzle determine the amount of pressure needed to extrude it through the nozzle (Mohammed et al., 2021). For example, during the 3D printing process, low viscosity may cause excessive material flow, whereas high viscosity may cause insufficient material flow. The nozzle size, shape, extruder speed, and printing pressure are other factors that can affect the 3D printing process (Vithani et al., 2019b).

**Fig. 4:**
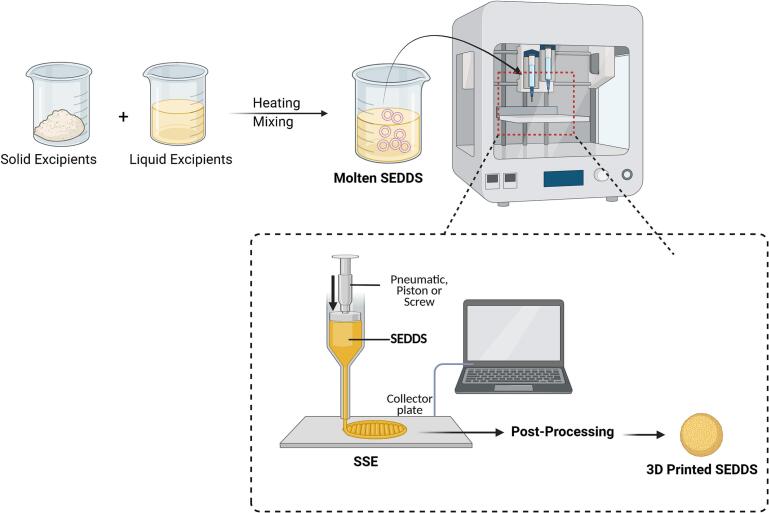
Schematic representation of the fabrication of 3D-printed self-emulsifying drug delivery systems (SEDDS) using semi-solid extrusion (SSE).

The final product is constructed by feeding the semi-solid material into the hot end of a 3D printer, where it is melted and deposited layer by layer. The intrinsic properties of the materials used in PAM enable them to function at lower temperatures, making them more suitable for thermosensitive formulations (Abdella et al., 2021). In contrast to the solid filaments or powders employed in traditional extrusion-based 3D printing, semisolid gels and pastes inherently possess lower melting points. Moreover, PAM systems were designed to minimise the heat exposure during the printing process. This precise temperature regulation ensures that the material remains within an optimal extrusion range, preventing exposure to excessive heat that could lead to degradation or changes in its properties (Dumpa et al., 2021). Fig. 4 illustrates the Pressure-Assisted Microextrusion method*.*

##### 4.1.2.1 Case study 1

**Fig. 5:**
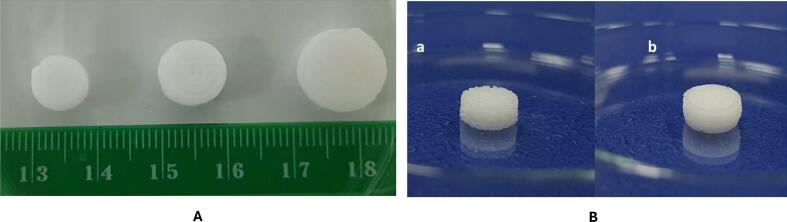
3D-printed self-nanoemulsifying tablets fabricated using SSE/PAM method: A) varying diameters (8 mm, 10 mm, and 12 mm). B) Rough, granular surface of tablet without continuous stirring (a), and smooth, uniform surface with continuous stirring (b). Figure reproduced with permission from (Algahtani et al., 2021).

Algahtani M. S al. highlighted the use of pressure-assisted microsyringe (PAM) extrusion-based 3D printing to develop a self-nanoemulsifying tablet of dapagliflozin propanediol monohydrate. A semi-solid paste was prepared using the fusion method, combining liquid phase components, caproyl 90, octanoic acid, and PEG 400, with solid-phase excipients, including poloxamer 188 and PEG 6000. The tablet geometry was designed using Autodesk CAD software, converted into an STL file, and processed into G-code using the Repetier software for printing. A Biobot 1 3D printer was used to fabricate circular self-nano-emulsifying tablets with different sizes (8 mm, 10 mm, and 12 mm) by extruding the semi-solid mixture (Fig. 5A). Scanning Electron Microscopy confirmed that dapagliflozin propanediol monohydrate was successfully encapsulated in the solid matrix of the self-nano-emulsifying tablets. An immediate-release profile was demonstrated for all tablet sizes (8, 10, and 12 mm), with more than 75 % of the drug being released in 20 min. The processing conditions, particularly mixing methods, are pivotal in ensuring extrudability, mechanical integrity, and print quality in PAM-based 3DP of lipid formulations. Inadequate or absent mixing leads to premature solidification of excipients within the syringe, producing tablets with rough and irregular surfaces. In contrast, continuous magnetic stirring generates a uniform semisolid paste, enabling consistent extrusion, smooth surface formation, and reproducible tablet morphology (Fig. 5B). The study recommends implementing continuous mixing of molten solid and liquid excipients prior to loading in the extruder syringe. These findings underscore the capability of PAM-based 3D printing to fabricate customised, rapid-release self-nanoemulsifying tablets, establishing a strong structure–process–performance correlation and demonstrating its promise for advanced drug delivery and enhanced bioavailability, although *in vivo* validation is still required. (Algahtani et al., 2021).

##### 4.1.2.2 Case study 2

A customised lipid-based suppository was developed using PAM to encapsulate different concentrations of lidocaine. Printable inks were developed by combining an oily component with a blend of surfactants. They were assessed for their physicochemical characteristics, along with their similarity to the functional performance of SNEDDS. A thixotropic composition of refined soybean oil, glyceryl distearate, and polyglyceryl-3 dioleate, marketed as Geloil™ SC, has been reported for its function and implications in enhancing the effectiveness of lipophilic drug delivery. The 3D printing conditions employed in this investigation were defined using an 18 G (1.27 mm) needle gauge, a layer height precision of 0.4 mm, and a structural infill density of 40 % with a concentric filling pattern. The suppository geometry was tailored with a total height of 20 mm, demonstrating a gradually expanding diameter from 5 mm at its base to 8 mm at a height of 15 mm, seamlessly transitioning into a 45° arc with a radius of curvature of 8.36 mm. The selected ink exhibited nanoscale emulsion droplets with a large surface area available for absorption. Suppositories containing lidocaine exhibited delayed drug release. These results are accelerating the development of 3D-printed suppositories that meet the needs of each patient and the treatment requirements (Chatzitaki et al., 2021).

However, the work lacks comparative drug release data and *in vivo* validation, limiting conclusions on therapeutic efficacy and clinical relevance. Overall, the study provides a promising proof-of-concept, but further investigations are needed to establish performance, safety, and scalability.

##### 4.1.2.3 Case study 3

Park and Kim demonstrated the use of SSE 3D printing to create solid suppositories of ibuprofen using SMEDDS. After heating, homogenising, and cooling to a semi-solid mass, a mixture of triacetin (oil), Gelucire 48/16 (surfactant), and tetraethylene glycol (co-surfactant) was 3D printed and formed into suppositories (50, 100, and 200 mg) without the need for moulding. The amorphous state of ibuprofen within the solid matrix was validated by DSC, PXRD, and FTIR, guaranteeing structural integrity, drug distribution, and quick disintegration. Compared with raw ibuprofen powder, *in vitro* dissolution experiments revealed a much higher initial drug release (Park and Kim, 2024). This study presented a promising application of SSE 3DP to produce SMEDDS-based suppositories of ibuprofen, achieving enhanced dissolution and potential improvement in rectal bioavailability. While the work demonstrates good physicochemical characterisation and content uniformity, it lacks *in vivo* validation and comparative studies with conventional suppositories, limiting conclusions on clinical efficacy.

##### 4.1.2.4 Case study 4

One study used extrusion-based 3DP to create multi-compartment 3D-printed tablets that included SNEDDS of glimepiride (GLMP) and rosuvastatin (RSV) for customised drug therapy for metabolic disorders. Gel formulations were prepared using 4 % HPMC, with SNEDDS-based gels incorporating RSV or GLMP, while non-SNEDDS gels were prepared by dispersing the drug directly in water and adding HPMC. A powder blend of Avicel, PVPK90, lactose, methocel, and Ac-Di-Sol was incorporated into the gels to form smooth, homogenous pastes, with excipients serving as adsorbents, insoluble filler, and disintegrant. HPMC-based pastes were 3D-printed layer-by-layer and dried into solid tablets using an optimised SNEDDS formulation based on curcumin oil (94.43 ± 3.55 nm droplet size). Shear-thinning rheology, a semi-porous structure for improved drug release, and uniform drug distribution were validated by characterisation tests. SEM analysis showed that SNEDDS-based tablets had a more homogenous, less porous surface compared with non-SNEDDS tablets, likely due to improved paste consistency and reduced water loss during drying. The internal structures reflected formulation differences, with SNEDDS incorporation producing a gel-like matrix with fewer voids, supporting enhanced uniformity and potentially improved drug release control. Rat pharmacokinetic tests revealed a notable increase in bioavailability (159.50 % for GLMP and 245.16 % for RSV) over commercially available tablets, while *in vitro* dissolution studies showed better drug release from SNEDDS-loaded tablets than from non-SNEDDS formulations. However, clinical validation and long-term stability studies are needed to fully confirm therapeutic advantages. (Ahmed et al., 2021).

### 4.2 Material jetting method

#### 4.2.1 Drop-on-demand (DOD) method

**Fig. 6:**
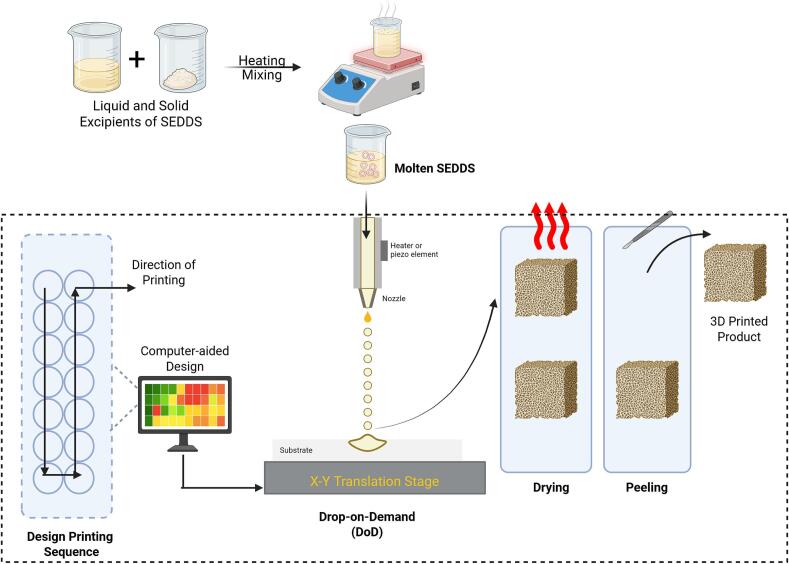
Schematic representation of the fabrication of 3D-printed self-emulsifying drug delivery systems (SEDDS) using Drop-on-Demand (DoD).

Material jetting involves selective deposition of material droplets onto a build platform using a print head (Vithani et al., 2019b). Drop-on-demand (DOD) inkjet printing is a type of MJ method with thermal and piezoelectric actuation mechanisms, most often used for controlling DOD print heads. The thermal print heads temporarily raise the temperature close to the resistor, which produces heat to create vapour bubbles that eject material droplets (Fig. 6). Localised heating can degrade thermolabile drugs, although this approach is limited to volatile solvents, making it less suitable for pharmaceutical applications (Shah et al., 2021). Piezoelectric print heads use piezoelectric elements, such as ceramics or crystals, that deform under electrical current to create pressure for ejecting liquid droplets. This method works with less volatile liquids at room temperature, making it more suitable for pharmaceutical applications (Rahmati et al., 2009).

DoD printing technology facilitates precise and controlled layering of active pharmaceutical ingredients (API) onto ingestible surfaces, including polymer films or tablet placeholders (Hirshfield et al., 2014). DoD offers enormous potential for customised doses because the dosage can be accurately and consistently adjusted to match a patient's recommended dose by combining the drop size and number of drops. The DoD system comprises a reservoir of materials, a precision positive displacement pump, xy-stage positioning, an integrated imaging and sensing unit, an air-based heating mechanism, multiple temperature-controlled heating elements, and temperature, pump, and stage movement controllers. The formulation containing the drug was placed in the material reservoir, and the precision pump regulated the ejection of droplets with precise size control through a nozzle. Droplet sizes typically range from 5 to 25 μL, depending on the nozzle dimensions, pump settings, and rheological properties of the formulation (Içten et al., 2017). An automated imaging system captures real-time images of falling droplets after ejection to determine their volume. After being deposited onto a chosen edible substrate, the droplet size can be changed using the nozzle configuration and pump parameters, allowing for individualised dosage modifications for each patient (Li et al., 2013). An array of melt-based formulations can be fabricated using the DoD. These formulations comprise a low-melting-point carrier combined with an active pharmaceutical ingredient that is blended and heated beyond the melting threshold of the mixture to generate hot melts. Different carriers can be utilised in the formulation of solid dispersions, depending on the properties of the solid state. The primary factors influencing the solid state are nucleation and crystallisation during melt cooling.

##### 4.2.1.1 Case study

Içten E, Nagy ZK, Reklaitis G V investigated Dropwise Additive Manufacturing of Pharmaceutical Products (DAMPP) as a novel approach to the production of melt-based solid oral dosage forms, specifically for self-emulsifying and amorphous drug delivery systems. Melt-based medication formulations can be precisely deposited onto inert substrates *via* DoD printing, allowing for highly accurate personalised dosing. In this study, the surfactant Gelucire 44/14 (10:90) was used to manufacture a 3DP formulation of celecoxib, a medication that is poorly soluble in water, and DAMPP was used for processing. After heating, the solution was applied to polymeric films and inert tablets, where it solidified into precise forms. X-ray diffraction and Raman spectroscopy confirmed that celecoxib was still present in the deposits in an amorphous form. The formulation's self-emulsifying qualities were demonstrated by the formation of a stable submicron emulsion (∼160 nm) upon contact with water according to Nanoparticle Tracking Analysis (NTA). Compared to crystalline celecoxib (∼22 % in 2 h), drug release from dissolution trials was much faster, reaching ∼75 % in 10 min. Nevertheless, despite the use of polymeric stabilisers, optical microscopy revealed that celecoxib recrystallised in the solution.

**Fig. 7:**
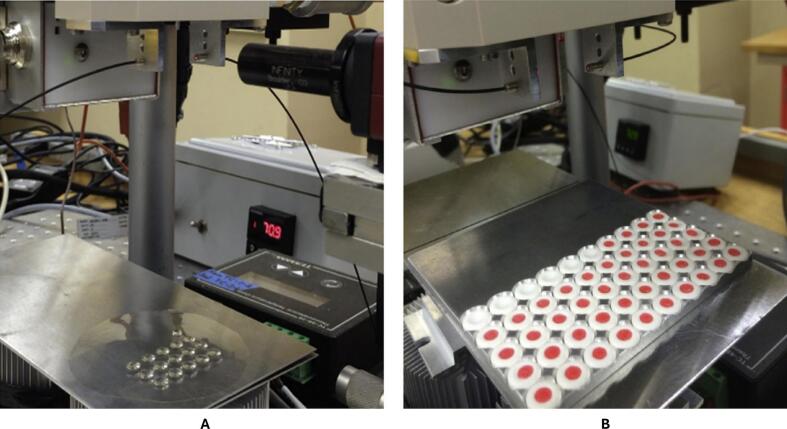
Fabrication of amorphous SEDDS melt-based formulations on hydroxypropyl methylcellulose (HPMC) films using a custom-designed DOD inkjet printer. Figure reproduced with permission from (Içten et al., 2017).

The advancement of DoD printing technologies has opened new avenues for designing amorphous and SEDDS. As illustrated by the preparation of SEDDS formulations on HPMC films (Fig. 7), substrate selection is crucial, as the surface chemistry, porosity, and morphology directly influence drug deposition, crystallisation tendencies, and dissolution behaviour. Excipient choice further dictates the performance of the final dosage form; high-melting lipids, such as Compritol, remain challenging under current prototype conditions, whereas surfactant-based systems, like Gelucire 44/14, facilitate both amorphisation and efficient self-emulsification. The integration of the DoD method into formulation development highlights not only the feasibility of precisely controlled drug deposition but also its potential for scalable, personalised medicine. Future research should focus on optimising substrate–excipient synergies and refining DoD hardware to enable robust, reproducible, and clinically relevant dosage forms.(Içten et al., 2017).

A summary of various 3D-printed SEDDS reported in recent literature is displayed in Table 1.

**Table 1:** Overview of reported 3D printed SEDDS formulation.

Sl. No.	3D Printing Method	Formulation	Drug	Outcome	Reference
1	Fused Deposition Modelling (FDM)	Bioactive self-nanoemulsifying hollow container tablet	• Curcumin • Lansoprazole	• SNEDDS produced droplets with a reduced size of 70.9 nm. • The polydispersity index (PDI) of the SNEDDS was within the range of 0.52–0.53 • Sustained drug delivery *via* floating and delayed-release properties • Multi-drug delivery	(Kulkarni et al., 2022)
		3D-printed single- and multi-compartment polymer scaffolds filled with dispersible SMEDDS	• Fenofibrate • clofazimine, • Lumefantrine • Halofantrine	• SMEDDS produced droplets with a reduced size of 5–10 nm. • Multi-drug delivery • Exhibited an approximately linear release, proportional to time in dispersion media • Formulation is gradually dispersed into the media based on the available surface area of the formulation	(Barber et al., 2021)
2	Pressure Assisted Microextrusion (PAM)	Self-nanoemulsifying tablet	Dapagliflozin Propanediol monohydrate	• Personalised Tablet • The average droplet size of the 3D printed self-nanoemulsifying tablets was found to be 104.7 ± 3.36 nm with a PDI value of 0.063 ± 0.024 • Immediate Drug Release Profile, with over 75 % of the drug released within 20 min.	(Algahtani et al., 2021)
		3D printed Suppositories	Lidocaine	• Personalised formulation • Droplet sizes in the range between 31.0 ± 8.4 nm and 42.7 ± 13.5 nm • Delayed Drug Release Profile • Drug release is largely governed by the extent of formulation erosion	(Chatzitaki et al., 2021)
		3D-Printed Suppositories	Ibuprofen	• Customised formulation • The average droplet size of SMEDDS was 65.91 ± 1.88 nm, with PDI 0.10 • Demonstrated stable stacking without visible damage • Exhibited initial higher drug release • Enhanced Solubilization and bioavailability	(Park and Kim, 2024)
		Multi-compartment 3D-printed tablets	• Glimepiride (GLMP) • Rosuvastatin (RSV)	• Multi-drug delivery • Exhibited a droplet size of 94.43 ± 3.55 nm • Higher drug release compared to no-SNEDDS formulations • GLMP and RSV demonstrated relative bioavailability of 159.50 % and 245.16 %	(Ahmed et al., 2021)
3	Drop-on-Demand (DOD) Method	Melt-based solid SEDDS	Celecoxib	• The mean particle size is found as 160.76 nm • On-demand production method for amorphous and self-emulsifying dosage forms • The 3DP-SEDDS achieved ∼70 % drug release within 10 min and plateaued at ∼75 %, corresponding to the crystalline solubility limit (1.1–1.5 μg/mL) • Enhanced solubility and bioavailability	(Içten et al., 2017)

## 5 Therapeutic and technological advantages of 3D-printed SEDDS

### 5.1 Development of personalised medicines

One of the numerous advantages of 3DP is that it streamlines the production process and allows the development of customised medications. Customised medication is the primary therapeutic and technological advantage of 3DP in the pharmaceutical industry. Despite the exploration of 3DP technologies for conventional dosage forms, including tablets (Khaled et al., 2014), capsules (Matijašić et al., 2019), and transdermal patches (Maurizii et al., 2023), the use of 3DP in producing customised SEDDS is still mostly overlooked. Customised medicine reshapes healthcare by moving away from conventional treatments and focusing on individual-specific approaches. This requires a thorough understanding of how genetic variations influence the risk of developing particular diseases (Brittain et al., 2017). Oral drug delivery systems, such as tablets, capsules, and liquids, have established themselves as patient-friendly and safe options; however, their therapeutic potential is still limited to personalising drug release profiles and dosing (Goyanes et al., 2015b). 3DP enables personalised production on an industrial scale or through distributed manufacturing, allowing drug carriers to be rapidly tailored to different shapes and doses to meet individual therapeutic requirements. A recent study investigated the viability of using PAM to produce lidocaine-free base-loaded suppositories, emphasising lipid ink systems that offer both biocompatibility and extrudability. GeloilTM SC, a thixotropic blend of refined soybean oil, glyceryl distearate, and polyglyceryl-3 dioleate, has shown promise in preserving the structural integrity required for suppository use while promoting uniform drug distribution. Lidocaine-loaded suppositories prepared from formulations with different lipid-to-surfactant ratios exhibited comparable physical and mechanical properties and variations in *in vitro* release kinetics, indicating their ability to modulate drug release behaviour by changing the composition of the formulation. These results highlight the potential of 3DP to develop personalised dosage forms that meet the demands of each patient (Chatzitaki et al., 2021). In a related study, PAM-based 3DP was used to fabricate dapagliflozin-loaded SNEDDS tablets for oral use. This approach effectively enhanced the solubility of dapagliflozin and permitted dimensional adjustments during printing to accommodate personalised dose requirements (Algahtani et al., 2021). Algahtani et al. introduced a clinically feasible approach to dispense SNEDDS formulations at individualised doses that can be prepared without specialised 3D printing expertise. This process uses an FDM-printed capsule shell design that can be filled by hand with a molten mixture. The shell provides improved stability and protection by encapsulating the material once it solidifies. This method allows simple single-unit or multi-unit dose filling with a conventional pipette in a single step. Cyclosporine A was chosen as the model drug owing to its narrow therapeutic index and poor water solubility. In post-organ transplant care, accurate dose modifications must be made to ensure therapeutic efficacy while lowering the risk of toxicity or graft rejection. This novel method combines the ability to dispense customised doses based on patient needs with improved drug solubility for better absorption (Algahtani et al., 2024).

### 5.2 Management of polypharmacy with combination medication therapy

Polypharmacy, the practice of prescribing numerous medications, presents several challenges, including decreased patient adherence and a higher likelihood of confusion. This primarily arises from the difficulty in managing multiple tablets and the complexity of the dosing regimens. Addressing therapeutic complexity can be accomplished through the combination of drugs that simultaneously target different disease pathways. Furthermore, the adoption of polypills allows for the co-formulation of multiple drugs, thereby minimising the number of required administrations and potentially enhancing patient compliance (Hatami et al., 2024).

The oral bioavailability of lipophilic drugs can be enhanced by co-administration of lipids in the form of food- or lipid-based formulations (Preeti et al., 2023). The ability to customise lipid-based formulations represents a significant advancement in the use of lipophilic drugs. Research on the extrusion of lipid formulations in the context of 3DP is lacking, although many physicochemical characteristics of lipids permit their extrusion at low temperatures (Vithani et al., 2019b). Combining 3DP technologies with SEDDS has opened new avenues for the development of polypill strategies. A lipid-based solid SEDDS is extruded and pelletized to create creamy spheroids (Abdalla and Mäder, 2007). According to recent studies employing formulations based mostly on polymers, it is feasible to generate 3DP oral dosage forms with several medications and various release characteristics (Goh et al., 2021) (Khaled et al., 2015). Markl et al. revealed that dual-compartment tablets with liquid lipids can be manufactured using a 3D-printed scaffold made from a dissolvable polymer (Markl et al., 2017). The potential of 3D-printed polymer scaffolds loaded with dispersible lipid formulations, known as polymer lipid hybrid (PLH) tablets, to regulate dispersion and drug release was examined in detail by Barber et al. Numerous scaffold designs, including single- and multi-compartment structures, were methodically evaluated in this study, with an emphasis on factors including exposed surface area, scaffold degradability, and asynchronous drug release. Multi-compartment circular scaffolds made up of six discrete “wedges” were 3D printed and individually filled with six different combinations of three drugs (clofazimine, lumefantrine, and halofantrine), utilising two different solid SMEDDS formulations as a proof of concept for polypharmacy applications. The adaptability of PLH was demonstrated by the asynchronous release of drugs. These results highlight the potential of 3D-printed PLH devices to improve spatiotemporal drug release and dose customisation, providing a viable approach to manage intricate regimens in patients requiring polypharmacy (Barber et al., 2021).

One significant advancement is the creation of hollow 3DP tablets that can incorporate SEDDS in a single dosage form, enabling the simultaneous administration of multiple drugs. Curcumin and lansoprazole, two lipophilic drugs, were encapsulated within SNEDDS and subsequently loaded into 3D-printed hollow tablets as a notable example of this methodology. HME-derived filaments made from Soluplus and hydroxypropyl cellulose were utilised to fabricate these hollow dosage forms, resulting in materials with optimal print fidelity, mechanical stability, and biocompatibility. SNEDDS formulations were carefully pipetted into the internal cavity of the printer to preserve the stability of the SEDDS and prevent leakage or degradation. Furthermore, adjusting the wall thickness can modify drug release kinetics and include programmed lag phases to regulate the release of multiple drugs over time (Kulkarni et al., 2022). The fundamental ideas of the polypill concept are mirrored in this approach, which provides dose personalisation, chronotherapeutic control, and a decreased pill burden. It is particularly important for populations with chronic conditions, such as diabetes, cardiovascular diseases, or post-transplant care, where polypharmacy is prevalent.

Ahmed et al. investigated the use of 3DP technology to fabricate complex multicompartment tablets incorporating self-nanoemulsifying formulations of glimepiride (an antidiabetic agent) and rosuvastatin (an antihyperlipidemic). Patients with diabetes are two to four times more likely to develop cardiovascular diseases than those without the disease. Integrated treatments involving both glycemic and lipid regulation have the potential to improve cardiovascular outcomes in at-risk patients. The pharmacokinetic performance of glimepiride and rosuvastatin was significantly improved in 3DP tablet formulations compared with that in commercially available products. Furthermore, these formulations allow dosage customisation based on individual therapeutic needs (Ahmed et al., 2021).

SSE-based 3DP was used to develop a multi-drug suppository containing budesonide and tofacitinib citrate, two anti-inflammatory agents, for the treatment of acute severe ulcerative colitis. This localised delivery approach significantly enhances therapeutic efficacy by concentrating drug action at the site of inflammation in the rectum and colon, and facilitates personalised dosing of drugs with limited aqueous solubility (Awad et al., 2023).

### 5.3 Fabrication of modified release solid SEDDS formulations

3DP facilitates the fabrication of solid SEDDS, including those with sophisticated complex modified release (MR) properties. MR formulations are designed to modulate the onset and/or site of drug release, thereby enhancing therapeutic efficacy and patient compliance in ways that conventional dosage forms cannot (Goyanes et al., 2017). Enteric-coated tablets are an example of MR formulations that delay the release of the drug until it has passed through the stomach. Oral formulations that are enteric (gastro-resistant) protect drugs from degradation in the stomach or target drug release into the intestines (Arafat et al., 2023). 3DP provides significant possibilities for the accurate and simple design of low-cost MR formulations. In a recent study, 3D printed cores were coated with the enteric polymer Eudragit® L100 in a fluid bed coater to create 3D printed enteric-coated budesonide tablets equivalent to two commercial formulations. Eudragit L100–55 filaments were developed and used to print the coating of 3D printed cores to offer enteric characteristics, thereby eliminating the need for fluid bed coating (Goyanes et al., 2015a). Shaqour et al. explored the possibility of applying 3DP in the development of patient-specific MR 3D-Printed Capsules Containing a Ketoprofen SNEDDS. A pomegranate seed oil–based SNEDDS of ketoprofen was used as a capsule-fill formulation. Hydroxypropyl methylcellulose phthalate: polyethylene glycol (HPMCP: PEG) and poly (vinyl alcohol) (PVA) were used to create 3D-printed capsules that were specifically designed for delayed release and pH sensitivity, respectively. This investigation highlighted the relationship between material properties and drug release behaviour, with polymer type and capsule thickness playing significant roles. Enhanced therapeutic activity also underscores the potential of 3DP in the formulation of MR-SEDDSs. According to the findings, drug release was pH-dependent and delayed, with zero-order release occurring at higher pH values. In addition, the thickness of the capsule influences its release rate (Shaqour et al., 2024). The poor aqueous solubility and variable absorption of glimepiride were addressed in a study by Ahmed et al., who developed glimepiride tablets by using three techniques: direct compression, liquisolid compaction, and SSE-based 3DP. An SNEDDS of glimepiride was developed and integrated into liquisolid, directly compressed, and 3D printed tablets. All formulations showed satisfactory physicochemical characteristics; however, variations in their surface features and internal structures were evident. The 3D-printed tablets exhibited a porous and tortuous matrix that facilitated controlled drug release. An *in vivo* pharmacokinetic investigation revealed that 3D printed tablets have higher bioavailability (121.68 %) than commercial formulations. These results highlight the potential of 3D printed solid SEDDS for developing MR formulations with improved biopharmaceutical performance (Ahmed et al., 2022).

### 5.4 Tending to pediatric and geriatric populations

Pediatric and geriatric patients frequently face difficulties with traditional oral drug delivery systems owing to swallowing challenges, inflexible dosages, and sensory preferences. Liquid formulations, such as SEDDS, are effective in enhancing the solubility and bioavailability of poorly water-soluble drugs and present several challenges in pediatric patients. Children are sensitive to unpleasant tastes and smells, which can result in poor adherence to medication. Liquid SEDDS often contain surfactants and cosolvents that may impart a bitter or oily flavour, making it difficult for children to tolerate. Additionally, the high concentrations of cosolvents used to maintain drug solubilization may be harmful to pediatric formulations. Most pediatric formulations utilise the same solubilization methods as those used in adults.

Although the technology remains the same, the product's physical form and presentation have been adapted to be more pediatric-friendly (Salunke et al., 2022). Another concern is that dosing accuracy is crucial, as children require doses adjusted based on their body weight and age, and ensuring precise measurement with liquids is challenging (Galande et al., 2020). Additionally, the risk of spillage, instability during storage, and the need for preservatives further complicate their use in the pediatric population (Haywood and Glass, 2013).

Polypharmacy is a common concern among the elderly population, who often require many medications for various conditions (Pazan and Wehling, 2021). Polypharmacy can be addressed using personalised pills created using the 3DP. Individuals with cognitive impairment, such as dementia, may struggle with drug adherence (Chippa and Roy, 2025). This challenge can be addressed through 3D printed dosage forms customised for time, date, and weekday information for each patient.

### 5.5 3D printed SEDDS: economic and logistical gains

A paradigm shift from traditional centralised drug manufacturing at large pharmaceutical factories to a more distributed model is driven by 3DP in the pharmaceutical industry. This decentralised strategy aims to manufacture medicines closer to the point of care, which could be in local pharmacies, hospitals, or even patients' homes. With such a change, mass customisation or on-demand drug manufacturing could eventually play a role in mass manufacturing (Zhu et al., 2020). The substantial reduction in logistical challenges and related expenses associated with drug storage and transportation is a major benefit of this decentralised approach (Pravin and Sudhir, 2018). Large libraries of printed medication formulations can be stored in digital repositories, which would enable medical professionals to send these files to secure cloud networks. Patients or local facilities could then produce medications as needed, thereby minimising delays, transport difficulties, energy use, and waste linked to centralised supply chains. Furthermore, this proximity to end-users allows for a more flexible reaction to treatment needs, allowing for individualised drug regimens tailored to specific physiological situations (Norman et al., 2017).

Despite the advantages of SEDDSs in increasing solubility and bioavailability, they often encounter handling, storage, and transportation issues due to their liquid nature and component sensitivity. On-demand 3D printing could offer a solution by enabling the localised manufacture of solidified SEDDS, enhancing their stability and ease of distribution (Uttreja et al., 2025).

This strategy also has obvious advantages in situations where conventional drug distribution is impossible, such as in remote or disaster-affected areas, poor areas, and even space missions. In emergency or resource-constrained scenarios, such as operating rooms or ambulances, on-site 3DP may provide a key means for rapid medicine production (Norman et al., 2017).

## 6 Challenges

The integration of 3D printing into the development of solid SEDDS, despite its advantages, is still limited by challenges related to technological capabilities, manufacturing formulations, safety standards, regulatory frameworks, and practical application in clinical pharmacotherapy (Vaz and Kumar, 2021).

### 6.1 Challenges in process, materials, and print quality

The 3D printing of SEDDS faces several significant obstacles related to the printing procedure, material characteristics, and product quality. From a process perspective, formulations that contain a large number of particles or high-viscosity ingredients, such as SSE and DoD, frequently cause nozzle clogging in nozzle-based systems, such as SSE and DoD (Eduardo and Ana, 2021). High-dose SEDDS and SNEDDS complicate the rheological properties of the feedstock, posing challenges for the extrusion process (Ganatra et al., 2024). The self-emulsifying nature and distinct viscoelastic behaviour of SEDDS make rheological characterisation more challenging (Biradar et al., 2009).

The benefits of SSE 3DP are clear, but they are still in the research and development phase and have drawbacks, such as the requirement to pinpoint important process variables and comprehend how printable materials behave. Analysis of the rheological and textural characteristics of various formulations is required. Determining optimised printing procedures often requires a trial-and-error method. The lack of comprehensive data on feedstock properties and materials with appropriate printability and rheological qualities also complicates the SSE (Aina et al., 2025). The high-temperature processing of FDM can cause degradation of heat-sensitive drugs and excipients, thereby limiting its application in SEDDS printing. The necessity of a pre-processing method, such as filament making, and the absence of suitable thermoplastic polymers, such as FDM polymers (PVA, Eudragit, and HPC), further restrict the application of FDM (Azad et al., 2020). In addition to being compatible with the drug, these materials must be biodegradable and suitable for use in the 3DP of SEDDS. Additionally, they should not generate toxic or harmful byproducts during or after processing (Lamichhane et al., 2019).

### 6.2 Safety challenges in 3DP of SEDDS

In every formulation development, safety must be ensured, and 3DP procedures must consider this. When exposed to heat, fusion, or extrusion, some materials may release harmful airborne particles that can irritate the skin or the respiratory tract. Therefore, adherence to standard operating procedures and stringent safety measures is required to reduce exposure to these toxic compounds. A draft guidance detailing the technical issues for regulating 3D-printed medical devices was produced by the FDA in 2017. The guidance mainly focuses on the design, production, and use of these devices. However, this advice might not be universally applicable to all 3D-printed items because safety and effectiveness evaluations are needed. Currently, there are no rules or criteria for 3D-printed formulations, despite the FDA having approved the first 3D-printed pill.

### 6.3 Technical challenges in 3D printing: from modelling to postprocessing

Modelling, slicing, printing, and post-processing are the four primary processes used in 3D printing. After establishing a printing model and slicing it to determine the printing path for each layer, the computer constructed a formulation with a complex structure based on the prefabricated model. Consequently, the modelling and slicing software must be updated frequently to meet the increasing printing standards as the complexity of structures evolves. Few software solutions have been purpose-built for 3D printing, and in DOP systems, the frequent start-stop action of the print head places a heavy strain on its stability. Multiple nozzles have been added and improved in extrusion-based 3DP methods such as FDM and SSE to handle different types of formulations. However, if the nozzles are not perfectly aligned, this can lead to an uneven distribution of the drug and an unstable product. This highlights the requirements for advanced machines, software, and control systems. In addition, excessive powder handling in DOP must be managed carefully to avoid waste and protect the health of workers (Cui et al., 2021).

### 6.4 Regulatory aspects

There is a growing expectation that people may be able to make their own drugs at home in the future, thanks to 3DP. Nonetheless, this possibility presents noteworthy regulatory obstacles, specifically regarding ensuring the security, effectiveness, and calibration of medications that are home-printed. Therefore, regulatory bodies such as the FDA will ultimately have to deal with how to monitor and manage access to digital medicine algorithms or “recipes” for personal use.

Although Spritam® (levetiracetam), the first 3D-printed medication, was approved by the FDA in August 2015, several issues remain to be addressed, including inconsistent product quality and mechanical instability (Azad et al., 2020). According to Alhnan et al., to meet the specific requirements of 3D-printed medications, the FDA may need to radically alter its traditional regulatory framework. The FDA is currently conducting a study on this new technology; however, it will likely take time to establish a comprehensive and uniform regulatory framework (Alhnan et al., 2016).

### 6.5 Challenges in production scalability

The application of 3d printing in the manufacturing of solid SEDDS holds great potential; however, it encounters obstacles, particularly in large-scale production. Traditional mass manufacturing remains predominant, and 3D printing is unlikely to replace it. Nevertheless, 3d printing offers substantial benefits to produce SEDDS. The integration of hot melt extrusion and 3d printing removes the need for downstream processes that are typical in current pharmaceutical manufacturing practices. Fused deposition modelling utilises low-cost printers and requires minimal postprocessing. Despite its benefits, 3D printing faces challenges in supplanting traditional industrial methods because its throughput is relatively low, owing to slower processing speeds. Additionally, 3DP SEDDS must follow the cGMP requirements set by the FDA (Azad et al., 2020). Table 2 demonstrates the various benefits and limitations of 3D printing methods used for the development of SEDDS.

**Table 2:** Advantages and limitations of 3D printing methods used for the development of SEDDS.

Sl. No	Method	Advantages	Limitations	References
1	Fused Deposition Method (FDM)	• Cost-effective and widely acceptable • Suitable for thermoplastic Polymers • Provides a solid carrier matrix for SEDDS • Stable solid SEDDS • Modified drug release solid SEDDS	• Not Suitable for heat-sensitive components • Risk of Drug degradation • Poor resolution • Additional procedures are required to smooth the surface of the solid product	(Patel et al., 2022)
2	Semisolid Extrusion Method (SSE)	• Ideal for thermolabile drugs loaded SEDDS • Personalised solid SEDDS • Capable of extruding SEDDS with semisolid or paste consistency • Easy Material Adaptation	• Post-processing required • Limited availability of compatible materials • Poor Precision • Chances of uneven drug distribution and poor emulsification of SEDDS	(Aina et al., 2025)
3	Drop on Demand Method	• Allows multiple materials • Multidrug loading for polypharmacy management • Dose customisation • High precision and resolution • Minimal wastage	• Limited to low-viscosity materials • Chances of nozzle clogging • Complex process and maintenance • Chances of poor print quality due to high lipid content • Complex optimisation and process control	(Vithani et al., 2019b)

## 7 Future perspectives

The future of 3D printing for the development of self-emulsifying drug delivery systems is highly promising for enhancing personalised medicine. 3D printed SEDDS formulations can be adjusted to individual patient demands in terms of dosage, drug release patterns, and even drug combinations, improving therapeutic efficacy and compliance. Furthermore, on-demand manufacturing of SEDDS at the point of treatment may lessen dependence on large-scale production facilities, providing greater flexibility and faster access to drugs.

Emerging materials that are compatible with both 3D printing and SEDDS are predicted to improve formulation stability and emulsification performance. Furthermore, advanced research can be carried out to identify the possibilities of including smart technologies such as sensors or stimuli-responsive components in 3D printed dosage forms, which may enable real-time monitoring or controlled release.

The integration of 3DP with SEDDS has tremendous potential for advancing pharmaceutical development. Regulatory agencies are anticipated to create more precise policies and procedures to ensure safety, effectiveness, and quality in the use of 3DP in the development of SEDDS. Advanced guidelines are required because of the special properties of 3DP SEDDS formulations, such as their special structures and customised designs, which may open the door for wider acceptability and commercialisation.

Designing and creating complex structures that are impossible with conventional production is one of the most interesting potential applications of 3DP. Novel three-dimensional structures, such as gradient systems, multilevel designs, or porous matrices, can greatly improve emulsification qualities and alter drug release profiles in the case of SEDDS. This creates new opportunities to target certain pharmacokinetic requirements and optimise treatment performance.

An efficient SEDDS formulation with minimal waste is made possible by the precise material deposition of 3D printing. By using fewer excipients and raw materials, this approach not only improves dose accuracy but also supports sustainable pharmaceutical manufacturing. The 3DP of SEDDS is in line with green manufacturing objectives, as the industry transitions to more environmentally friendly processes.

## 8 Conclusion

In summary, the combination of 3D printing and SEDDS is an innovative technology that has expanded pharmaceutical formulation development. This groundbreaking technique provides unique possibilities for personalised, efficient, and patient-centred drug delivery applications. Several 3DP methods, including fused deposition Modelling (FDM), Semi-Solid Extrusion (SSE), and drop-on-demand (DoD) printing, have demonstrated substantive capability to produce SEDDS-based dosage forms. However, while these methods offer distinct advantages, they overcome several challenges, including material compatibility, printability and rheology issues, post-processing requirements, regulatory and quality assurance hurdles, scale-up, and commercialisation. Despite these challenges, the 3DP of SEDDS offers control over the geometry, structure, and drug distribution. A more effective and possibly environment-friendly SEDDS production process is promoted using 3DP, which also reduces the need for additional excipients and complex processing procedures. With the ongoing advancements in material science, printer technology, and regulatory standards, the 3DP of SEDDS is poised to become a game-changing approach in drug research and personalised medicine.

## CRediT authorship contribution statement

**Induja Govindan:** Writing – review & editing, Writing – original draft, Visualization, Formal analysis, Conceptualization. **Anjana A. Kailas:** Resources, Formal analysis, Data curation. **K.A. Abutwaibe:** Visualization, Data curation. **Thamizharasan Annadurai:** Visualization, Data curation. **Ujwala Achar:** Resources, Data curation. **Anup Naha:** Validation, Supervision, Project administration, Conceptualization. **Srinivas Hebbar:** Validation, Supervision, Project administration.

## Source of funding

This work was supported by the Intra Mural Fund from the Manipal Academy of Higher Education, India.

## Declaration of competing interest

The authors declare that they have no known competing financial interests or personal relationships that could have appeared to influence the work reported in this paper.

## Data Availability

No data was used for the research described in the article.
